# Supplementary material for: The ER membrane complex (EMC) can functionally replace the Oxa1 insertase in mitochondria
Source: PLoS Biol. 2022 Mar 1;20(3):e3001380. doi: 10.1371/journal.pbio.3001380 (PMC8887752; doi:10.1371/journal.pbio.3001380)

Fig. 2b)

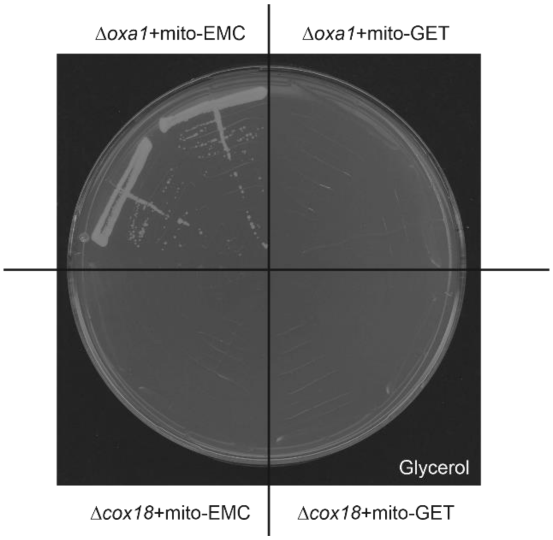

Δ*oxa1*+mito-EMC
Δ*oxa1*+mito-GET
Glycerol
Δ*cox18*+mito-EMC
Δ*cox18*+mito-GET

Fig. 2c)

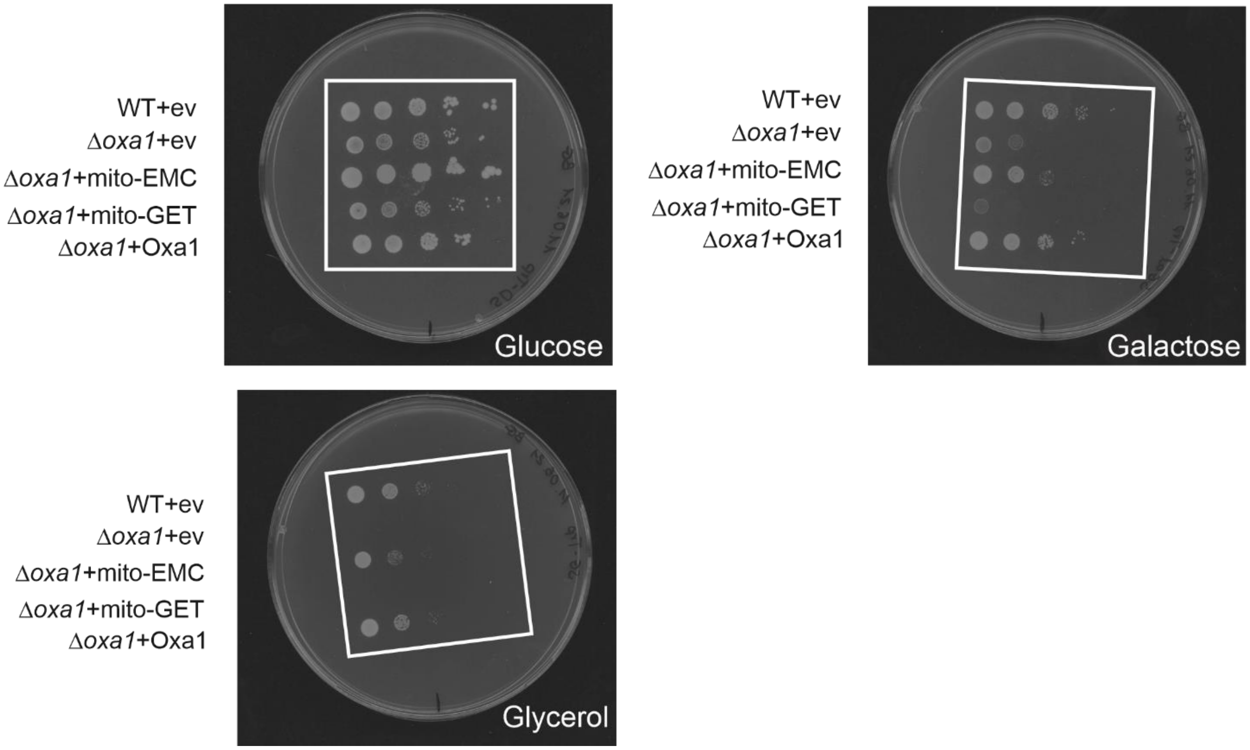

WT+ev
Δ*oxa1*+ev
Δ*oxa1*+mito-EMC
Δ*oxa1*+mito-GET
Δ*oxa1*+Oxa1
Glucose
WT+ev
Δ*oxa1*+ev
Δ*oxa1*+mito-EMC
Δ*oxa1*+mito-GET
Δ*oxa1*+Oxa1
Galactose
WT+ev
Δ*oxa1*+ev
Δ*oxa1*+mito-EMC
Δ*oxa1*+mito-GET
Δ*oxa1*+Oxa1
Glycerol

Fig. 2e)

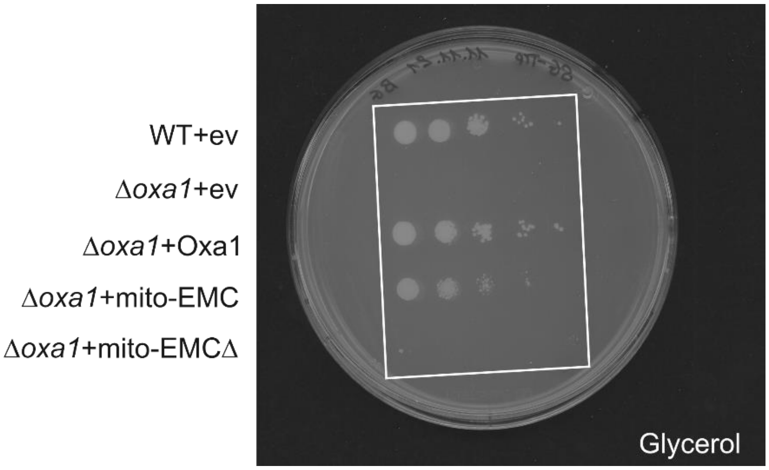
WT+ev
Δ*oxa1*+ev
Δ*oxa1*+Oxa1
Δ*oxa1*+mito-EMC
Δ*oxa1*+mito-EMCΔ
Glycerol

Fig. 2f)

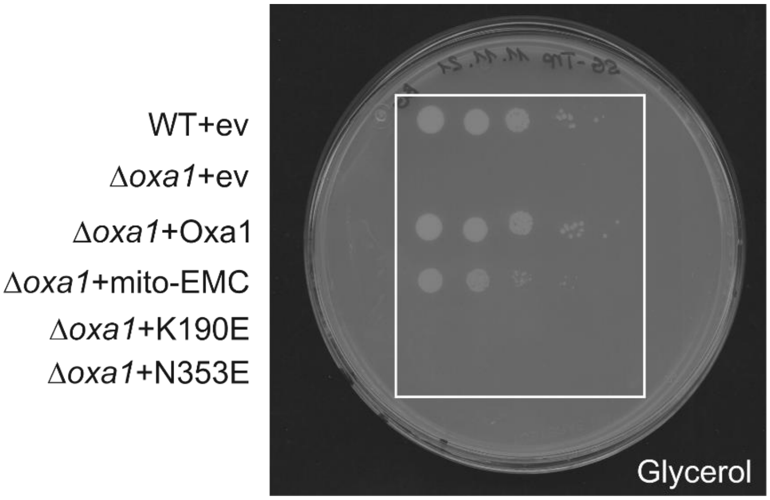
WT+ev
Δ*oxa1*+ev
Δ*oxa1*+Oxa1
Δ*oxa1*+mito-EMC
Δ*oxa1*+K190E
Δ*oxa1*+N353E
Glycerol

Fig. 3b)

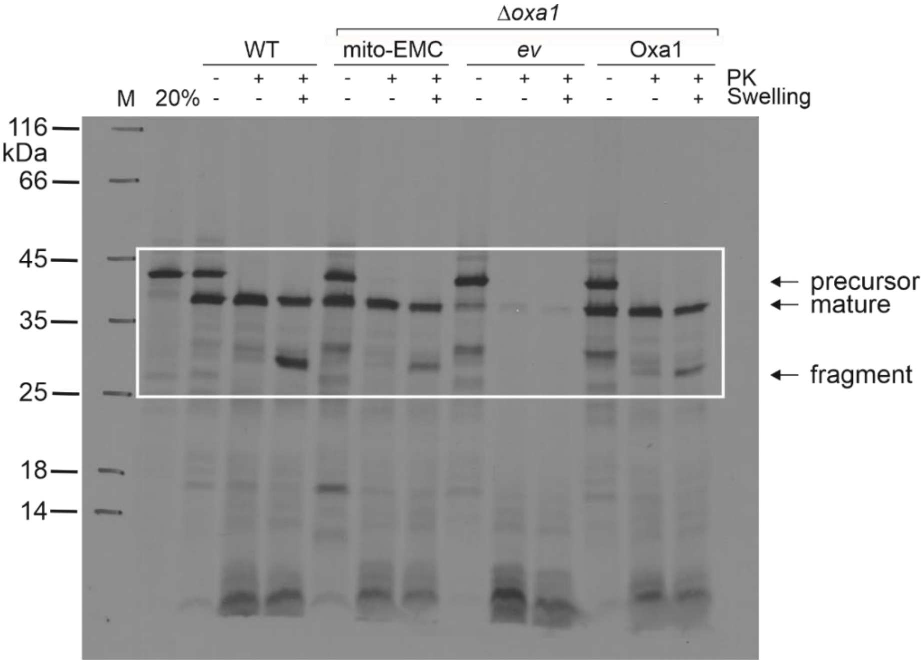
Δoxa1
WT
mito-EMC
ev
Oxa1
PK
Swelling
M
20%
116
kDa
66
45
35
25
18
14
precursor
mature
fragment

Fig. 3c)

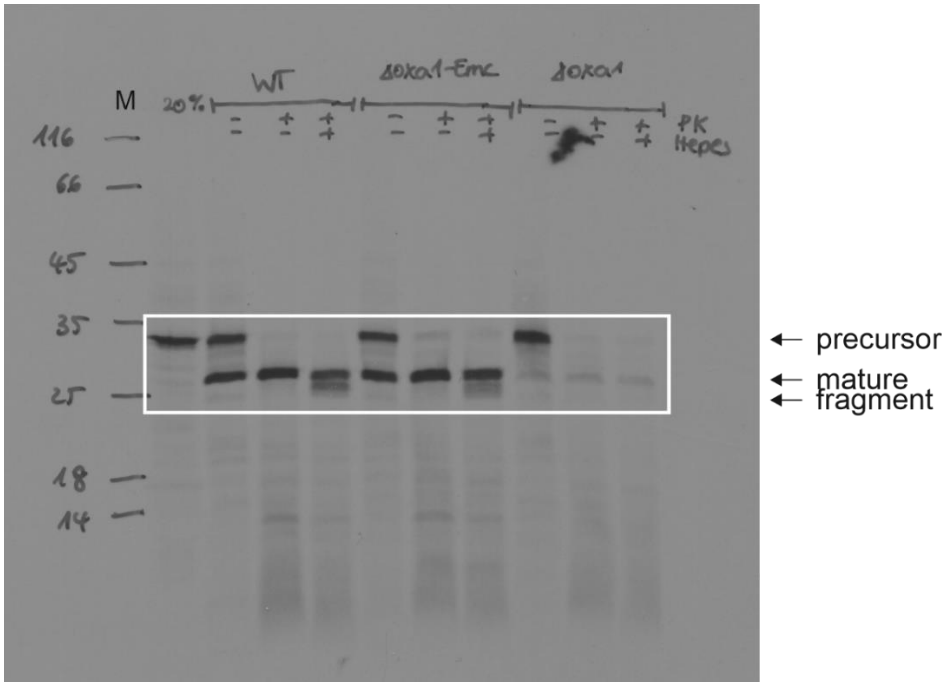
M
20%
WT
PK
116
66
45
35
25
18
14
precursor
mature
fragment

Fig. 3d)

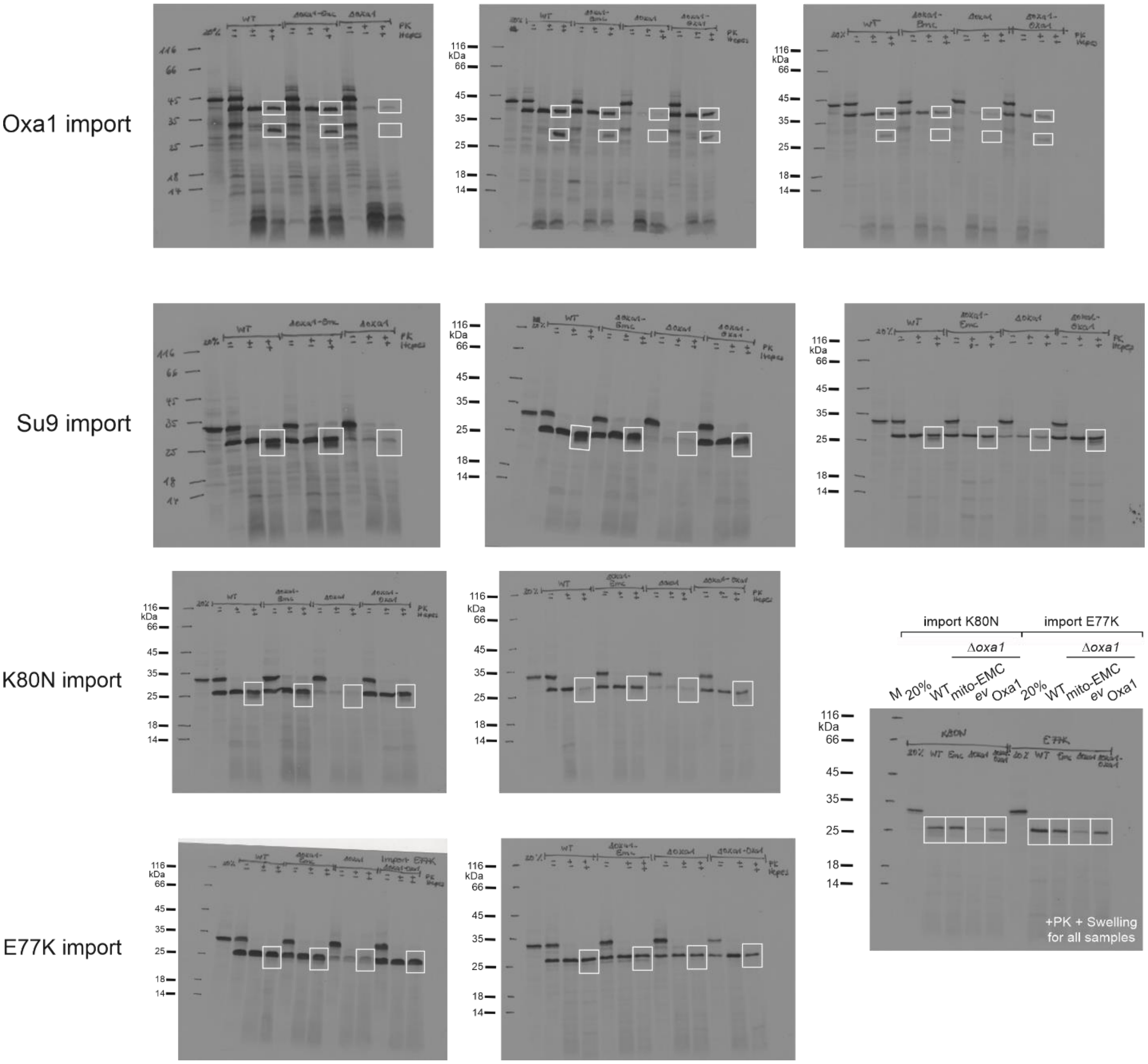

Oxa1 import
Su9 import
K80N import
E77K import
116
kDa
66
45
35
25
18
14
import K80N
import E77K
Δoxa1
Δoxa1
M 20% WT mito-EMC ev Oxa1 20% WT mito-EMC ev Oxa1
+PK + Swelling
for all samples

Fig. 4a)

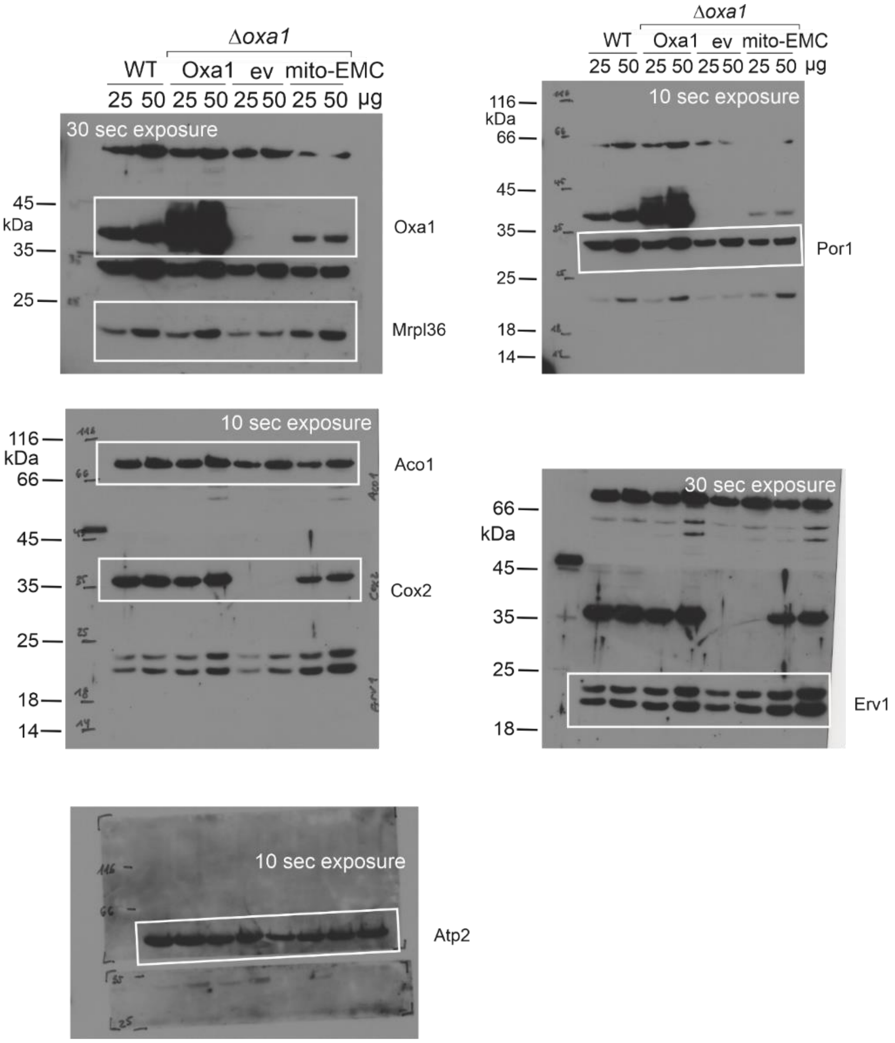

Δoxa1
WT Oxa1 ev mito-EMC
25 50 25 50 25 50 25 50 µg
30 sec exposure
10 sec exposure
Oxa1
Mrpl36
Por1
Aco1
Cox2
Erv1
Atp2
116
66
45
35
25
18
14
kDa

Fig. 4b)

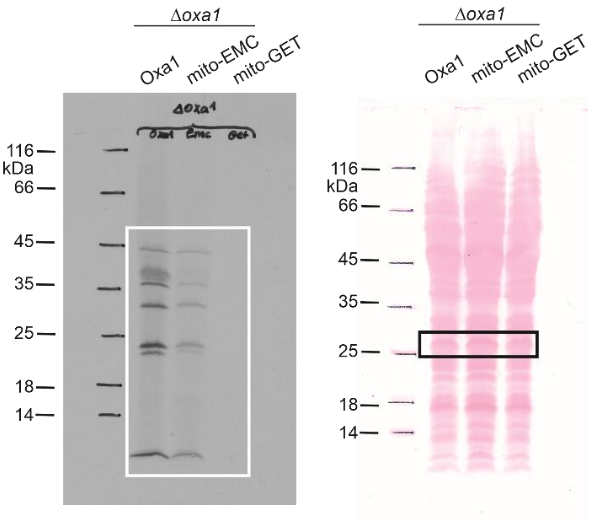

Δoxa1
Oxa1
mito-EMC
mito-GET
116
66
45
35
25
18
14
kDa

Fig. 4c)

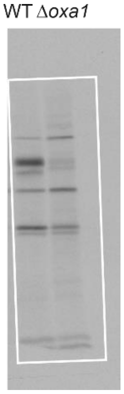

Fig. 4d)

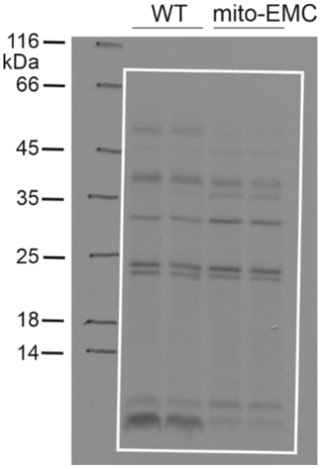

Fig. 4e)

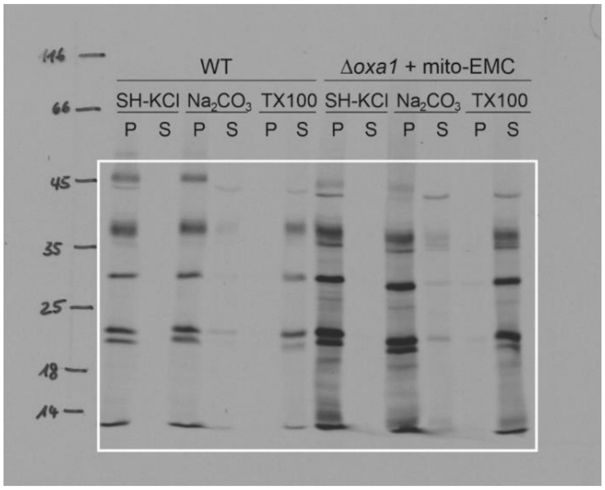

Fig. 4f)

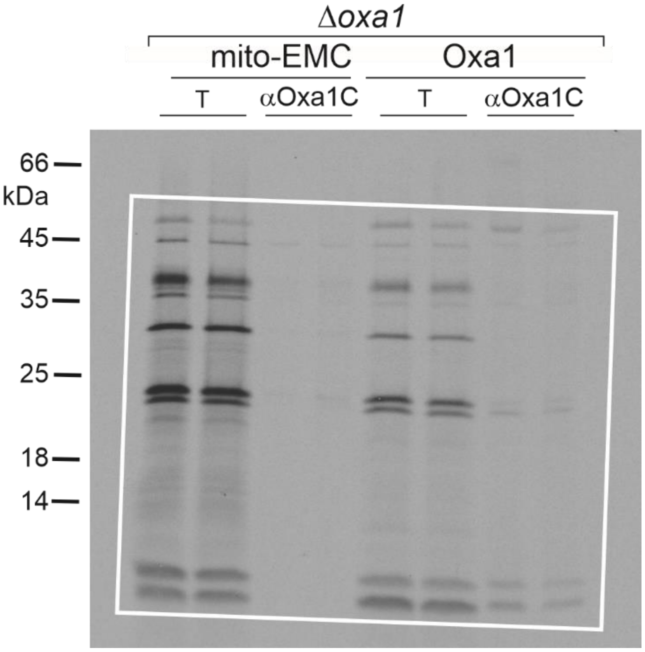
Δoxa1
mito-EMC
Oxa1
T
αOxa1C
T
αOxa1C
66
kDa
45
35
25
18
14

Fig. 4g)

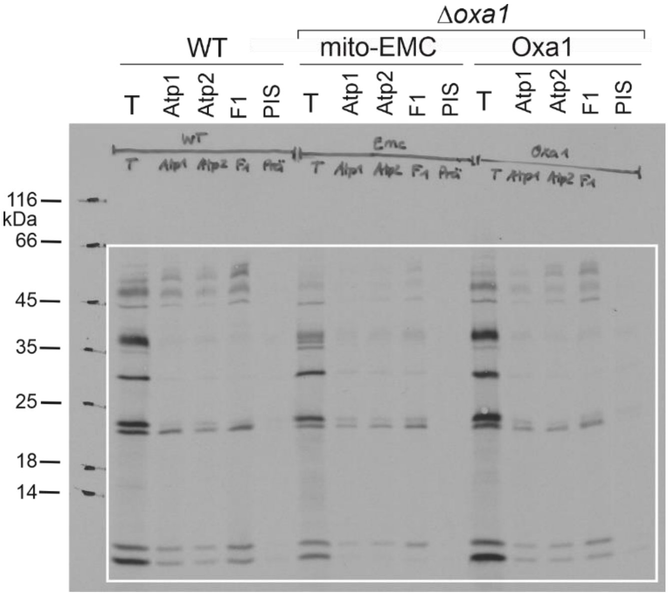
Δoxa1
WT
mito-EMC
Oxa1
T
Atp1
Atp2
F1
PIS
T
Atp1
Atp2
F1
PIS
T
Atp1
Atp2
F1
PIS
116
kDa
66
45
35
25
18
14

Fig. S2a)

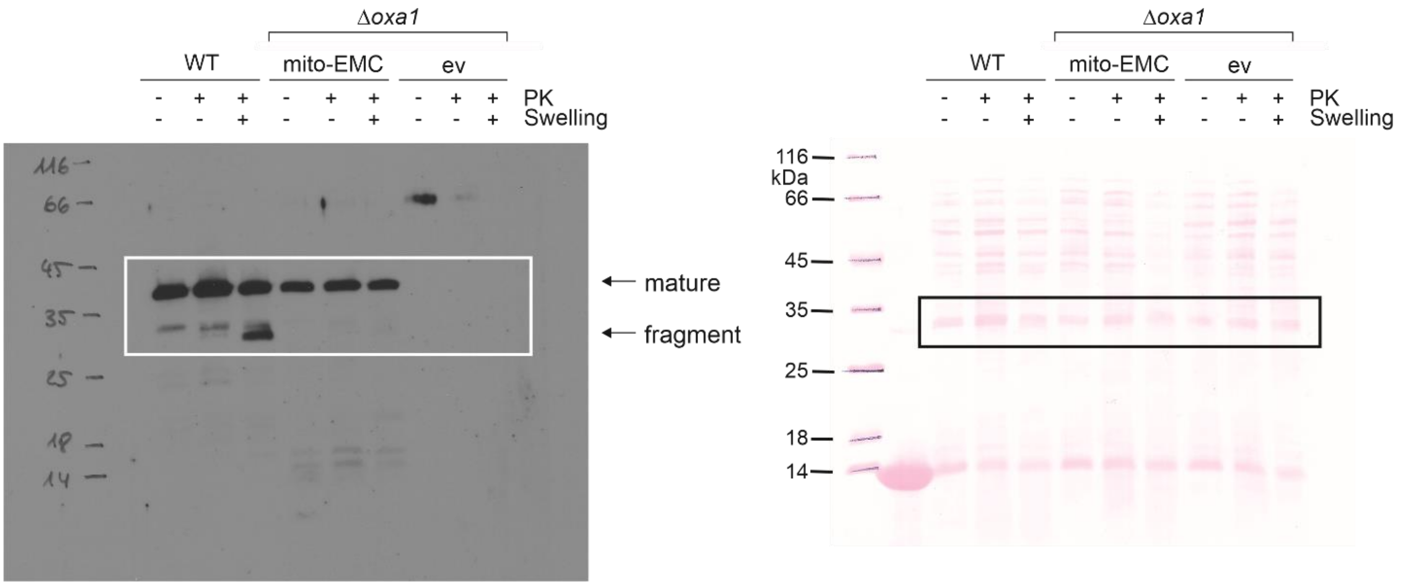

Fig. S2b)

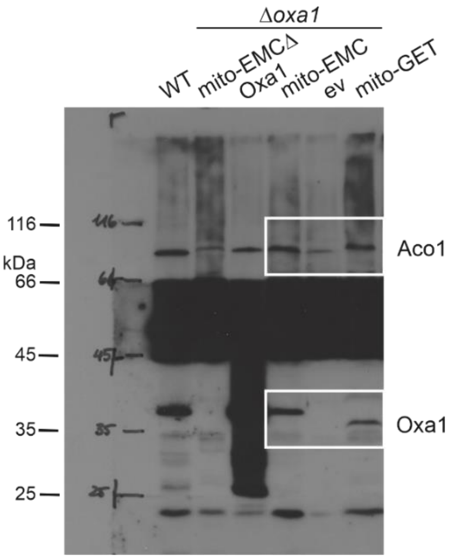

Fig. S2c)

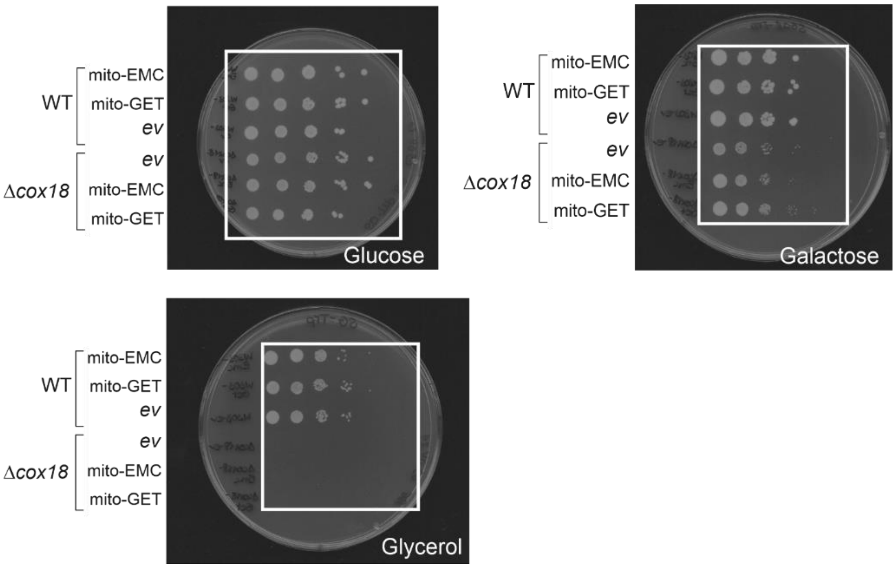

Fig. S2d)

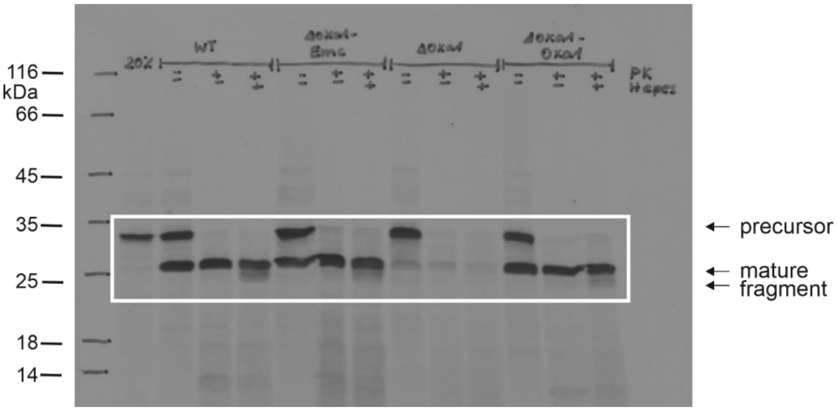

Fig. S2e)

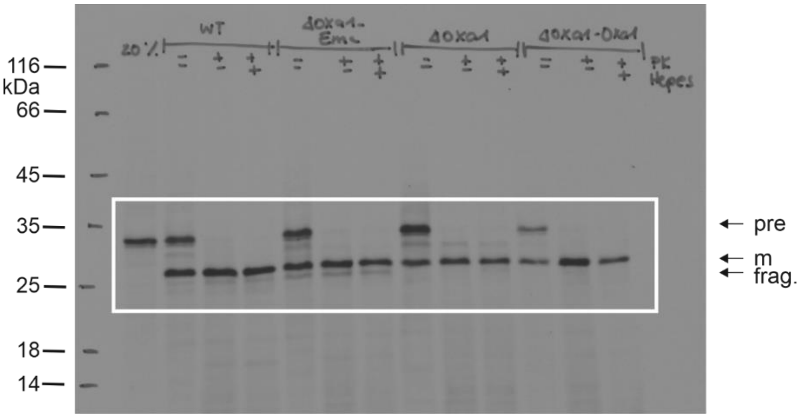

Fig. S3a)

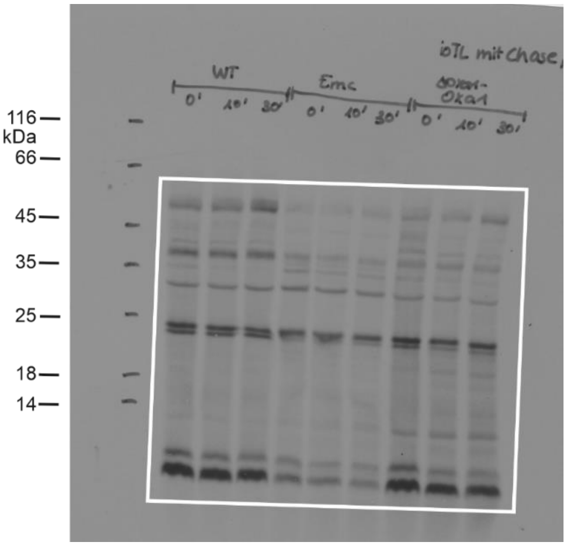

Fig. S3b)

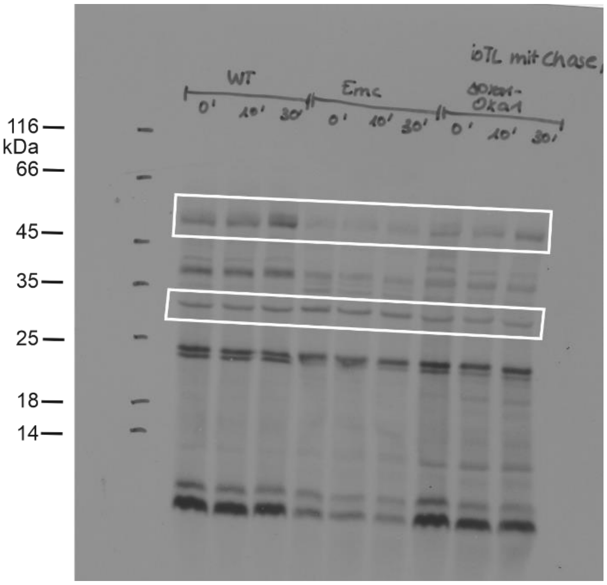

Supplement: S5 Data — Original scans and images of agar plates are shown here that were used to prepare the figures of this study. (PDF) [file pbio.3001380.s008.pdf]
