# Supplementary material for: The ER membrane complex (EMC) can functionally replace the Oxa1 insertase in mitochondria
Source: PLoS Biol. 2022 Mar 1;20(3):e3001380. doi: 10.1371/journal.pbio.3001380 (PMC8887752; doi:10.1371/journal.pbio.3001380)
Supplement: Attachment — Submitted filename: renamed_36fb8.pdf [file pbio.3001380.s009.pdf]

Specific points of the referees

Reviewer #1: Major Comments The YidC/Oxa1/Alb3 family of proteins is crucial for membrane insertion in bacteria, mitochondria, and chloroplasts, respectively. Intriguingly, possible homologs of these proteins have recently been discovered in the ER as determined by solving the structures of the ER transmembrane complex (EMC) and the ER guided entry of tail anchor proteins (Get) complex. The structures showed the two Get1-Get2 subunits and the core subunits Emc6-Emc3 of the EMC shared overall similarity to the solved structure of the bacterial YidC.

In this paper submitted by Herrmann and coworkers, the authors test whether the Emc6-Emc3 protein and Get2-Get1 could take over the function of Oxa1 or Oxa2 in mitochondria. Specifically, the constructs had an amino-terminal mitochondrial targeting sequence, a linker region fusing the two subunits (either Get2-Get1 or Emc6-Emc3) and the Oxa1 derived C-terminal (matrix) ribosomal binding domain. Interestingly, the authors showed that the Emc6-Emc3 (but not Get2-Get1) could functionally replace Oxa1 and complement growth in an Oxa1 deletion yeast strain. Neither fusion construct restored growth to the Oxa2 deletion strain. The data showed the mitochondrial inner membrane localized Emc6-Emc3 construct could insert the Oxa1-dependent Oxa1 protein and Cox2. This suggested that Su9 (1-112) was also inserted although the raw data was not entirely convincing (see below). While the Emc6-Emc3 construct did insert some of the known Oxa1 substrates, they conclude that it also fails to function in catalyzing (at least efficiently) the assembly of the Atp9 oligomer.

Overall, the paper provides clear data that the core subunits Emc6-Emc3 can replace some of the functions of the Oxa1 insertase in mitochondria. Thus it supports the authors' main conclusion that ER protein insertion is functionally conserved to the insertion mechanism in bacteria and mitochondria.

We thank the referee for her/his positive comments to our study. We are also very grateful for the careful proof-reading and the valuable comments which clearly improved our manuscript.

Other Comments

1.In Figure 3C, the data seems to show little export of the N-tail of Su9 (1-112) with the mito-EMC facilitating insertion while the quantitation of the data presented in 3D suggests that export is quite good. I would recommend showing the replicates of the data used to calculate the bar graphs in 3D in the supplementary data section.

We replaced figure 3C as suggested by an experiment in which the ability of mito-EMC to insert Su9-(1-112) is better visible.

2. In Figure 4A and 4B, there is a band at the position of the Cox2, which the authors indicate is the Cox2 protein, which I assume is the mature form of Cox2. It is hard to judge if there is any precursor Cox2 in the mito-EMC lanes since the predicted position is not indicated. Maybe a darker gel image in the supplementary section would be helpful to rule out there is any precursor. Could the authors indicate where the Cox2 precursor would be located?

Thank you for this suggestion. Indeed, the precursor form of Cox2 (which just migrates above the mature form) is often difficult to see as it is occluded by the Cox1 signal. To illustrate this better, we now added a new panel showing the translation products in WT and *Δoxa1* mitochondria (novel Fig. 4C). Moreover, we added a novel figure showing the fractions from carbonate extraction in the different mutants. Thereby, the precursor of Cox2 is separated from the mature Cox2 and from Cox1 since it is largely extractable. As shown in the novel Fig. 4E, some precursor of Cox2 accumulates in the mito-EMC mitochondria, however, most of the Cox2 is matured. As a third novel data item, we added a Western blot showing that Cox2 accumulates at steady state levels in mito-EMC cells whereas no Cox2 is detected in *Δoxa1* cells (novel Fig. 4A).

3. The authors state on page 8 "immunoprecipitation of ATPase complexes with antibodies against Atp1, Atp2 or the F1-part of the ATPase copurified ATP6, ATP8, and ATP9 in Oxa1-containing mitochondria, but not in those of the mito-EMC mutant." I recommend being more clear in this statement because in the mito-EMC mutant samples, ATP6 and ATP8 are found but not ATP9. Only ATP9 is missing.

We now corrected the sentence and write: "Moreover, immunoprecipitation of ATPase complexes with antibodies against Atp1, Atp2 or the F1-part of the ATPase copurified Atp6, Atp8 and Atp9 in Oxa1-containing mitochondria, whereas in mitochondria of the mito-EMC mutant, only Atp6 and Atp8, but no Atp9 was coisolated". We thank the referee for the careful proof-reading!

The authors also confirmed there is diminished ATPase activity (of the F1Fo ATPase) in mito-EMC mitochondria. However, there is still 50% of the ATPase activity when compared to Oxa1 mitochondria. This seems contradictory with the finding that the Atp9 oligomer is not formed in mito-EMC mitochondria. If no ATP9 oligomer forms, should there be no ATPase activity or is the ATPase activity expected to only be reduced? Please comment on this.

We added a further sentence to point out this aspect more clearly: "However, it should be noted that despite the Atp9 defect of the mito-EMC strain, still about 50% of the normal ATPase activity accumulated in the mutant explaining why this mutant efficiently grows on non-fermentable carbon sources."

This is also in line with previous studies. For example, Rosemary Stuart's group described before the Oxa1 dependence of ATPase assembly in the following way: "It is important to note, however, that although strongly reduced in levels, assembled and enzymatically functional ATP synthase complexes can still be detected in the Δoxa1 mitochondria, suggesting that a low level of Oxa1-independent ATP synthase assembly can occur." (L. Jia, M.K. Dienhart, R.A. Stuart, Oxa1 directly interacts with Atp9 and mediates its assembly into the mitochondrial F1Fo-ATP synthase complex, Molecular biology of the cell 18 (2007) 1897–1908.)

4. On page 3 and 4, it is stated that the "YidC monomeric proteins serve as an enzyme that accelerate the spontaneous (though often inefficient, slow and non-productive) partitioning of hydrophobic segments into the lipid bilayer." This is awkward. Obviously, YidC catalyzes insertion but then it is stated there is spontaneous partitioning. Please clarify. Also, YidC membrane insertion can be very efficient; I would omit the phrase "though often inefficient, slow and non-productive".

We rephrased the sentence and now write: "Several YidC structures were published recently which suggest that these monomeric proteins accelerate the partitioning of hydrophobic segments into the lipid bilayer"

5. Page 7 (second sentence from top). After the phrase "Oxa1-mediated insertion efficiency" add "of protein substrates". Also, it should be clear that the charge is added to the intramitochondrial space region of Su9 (1-112).

We rephrased the sentence which reads now: "Previous studies have shown that the Oxa1-mediated insertion efficiency is dependent on the negative charge in the region of protein substrates that are translocated to the intermembrane space and that positively charged regions are not exported by Oxa1".

The description of Fig. 3D was updated and now reads: 'Insertion efficiencies from three biological replicates of Oxa1, Su9-(1-112)-DHFR and two point mutations in Su9(1-112)-DHFR leading to a more negative (K80N) or positive charge (E77K) of its intramitochondrial space region were quantified.'

6. In Figure 4A, lower panel, state what the pinkish bands are?

We added an explaining sentence: The pinkish signals on the bottom show Ponceau S-stained protein bands verifying equal loading.

In the 4(C,D) Figure legend, add "onto the gel" after the word "loaded". Should be "loaded onto the gel."

We changed this as suggested.

Reviewer #2: Membrane protein biogenesis relies on different cellular machineries including pore-forming translocons as well as insertases without an evident pore. The mitochondrial inner membrane (IM) contains Oxa1 as an insertase for IM protein insertion from the matrix side. In this manuscript, Güngör et al. report a surprising observation that Emc6 and Emc2, components of the ER insertase EMC, can take the role of Oxa1 when they are expressed as a fused protein together with the Oxa1 C-terminal segment for mitochondrial ribosome recognition and targeted to the mitochondrial IM. The in vitro import and in vitro mitochondrial translation experiments suggested that this artificial fusion protein, mito-EMC, facilitated the insertion of nuclear-encoded Oxa1 and Su9(1-112), and mitochondrially encoded Cox2 into the IM. The successful substitution of the Oxa1 insertase function with mito-EMC should require integration of mito-EMC in the IM

with correct membrane topology, acquiring the correct functional structure of mito-EMC in the IM, correct recognition of Oxa1 substrates by mito-EMC, correct binding of mito-EMC with mitochondrial ribosomes to receive its substrates etc. Thus, to make this provocative finding more convincing, the following points should be clarified.

We agree with the referee that the observed complementation is surprising and thank her/him for the overall positive evaluation.

1. Successful membrane topology formation of mito-EMC is not easily expected, considering the difference in lipid compositions between the ER membrane and IM. Therefore, at least membrane topology, i.e. membrane integration and orientation, of mito-EMC, as expected in the IM should be assessed.

Following the suggestion of the referee, we performed a mitochondrial fraction experiment in which we opened the outer membrane by hypotonic swelling before proteinase K was added. By this treatment, protein regions are degraded which are exposed to the intermembrane space. Protease treatment produced a characteristic fragment from Oxa1 (which has an IMS-exposed N-terminus) but not from mito-EMC. This is consistent with the proposed topology of mito-EMC in the inner membrane. This result is now shown in the revised version as a novel figure panel (Fig. S2A). We thank the referee for her/his suggestion.

In addition, we performed carbonate extraction experiments with Oxa1 and mito-EMC. Unfortunately, both proteins form insoluble aggregates once released from membranes. Thus, they even accumulate in the pellet fraction after membrane solubilization with Triton X-100. We show the results of such an experiment here for inspection of the referee. Since this precludes any conclusions from carbonate extraction experiments on the topology of Oxa1 and mito-EMC, we decided not to show this experiment in the manuscript.

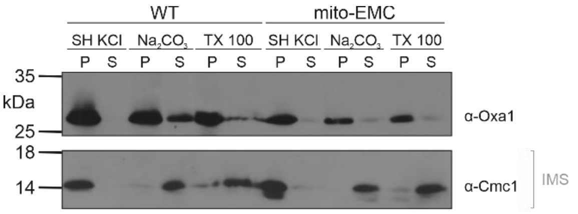

Figure for referee 2

Mitochondria were sonicated in the presence of 150 mM KCl, treated with 100 mM sodium carbonate, or lysed with 0.5% Triton X-100. Soluble (S) and pellet (P) fractions were separated by centrifugation (10 min, 18,000 rpm, 4°C) and analyzed by Western blotting. Cmc1 is a membrane-associated IMS protein shown for control. Please note that Oxa1 and mito-EMC are always found in the pellet fraction, even after complete solubilization of the membranes with Triton X-100.

2. Insertion of the Oxa1 substrates into the IM by mito-EMC should be tested by carbonate extraction, too, in addition to the protease clipping assay.

We now followed the suggestions of the referee and performed carbonate extraction experiments of mitochondrial translation products. Thereby we found that after synthesis

in WT mitochondria, Var1 is the only translation product that winds up solely in the carbonate supernatant. Since Var1 is the only translation product that is no membrane protein, this result was expected. In mito-EMC mitochondria, again all membrane proteins are found predominantly in the carbonate pellet, consistent with their insertion into the inner membrane. Interestingly, here small levels of the precursor form of Cox2 are observed in the mito-EMC sample which are extracted with carbonate just like Var1. This experiment nicely confirms our conclusions about the insertase activity of mito-EMC and is now shown as novel Fig. 4E.

3. To confirm that the observed insertion of the Oxa1 substrates relies on the mito-EMC function, the effect of the functional mutation in the EMC, such as Emc3(K26L) should be tested.

We thank the referee for this suggestion. We introduced two different point mutations in the mito-EMC protein at essential positions. Indeed, these variants were not able to take over the insertion function of Oxa1. This experiment is now shown as novel Fig. 2F.

4. The authors claimed that Emc3 and Emc6 form a minimal insertase unit that is able to promote protein insertion into a lipid bilayer. Then does expression of Emc3 and Emc6 rescue the deletion of the EMC in the ER membrane? In addition, can Oxa1 and Su9 mutant precursor proteins that lack their presequences be inserted into the EM membrane when the EMC deletion is rescued by Emc6-Emc2 in the ER membrane?

It is an interesting suggestion to test whether the Emc3-Emc6 fusion protein also would be functional in the ER membrane and, if so, which spectrum of substrates this minimal machinery would be able to deal with. However, we felt that this project would be beyond the scope of this study. Even if this sounds like a simple experiment, it would require thorough optimization and a number of controls, for example to make sure that the Emc3-Emc6 fusion in the ER is stable and not degraded by ERAD. Thus, it was not possible to follow this suggestion within the time frame of three months which PLOS Biology granted us. We also felt that this experiment, though certainly interesting, is not directly relevant for our conclusion, regardless of whether the Emc3-Emc6 fusion was functional in the ER or not.

5. The requirement of the Oxa1 C-terminal segment for mitochondrial ribosomes should be experimentally tested by comparing the function of mito-EMC with and without the Oxa1 C-terminal segment.

We thank the referee for this suggestion. We made a version of the mito-EMC protein that lacks the C-terminal matrix domain of Oxa1. Indeed, this variant is not able to take over the insertion function of Oxa1. This was expected as a C-terminally truncated version of Oxa1 that lacks the ribosome-binding domain fails to insert mitochondrial translation products into the inner membrane (Jia et al. EMBO J. 22, 6438f; Szyrach et al. EMBO J. 22, 6448f;). This experiment is now shown as novel Fig. 2E.

6. If mito-EMC is functional as an insertase in the IM, can EMC substrates artificially containing a mitochondrial presequence be inserted into the IM from the matrix side after import into the mitochondrial matrix?

Again, we felt that this suggestion is interesting, however, it is not as simple as it might sound. The hydrophobicity of transmembrane domains of ER proteins is much higher than that of nuclear encoded mitochondrial proteins. Thus, the import of these proteins would most likely result in the lateral integration of these proteins from the TIM23 complex into the inner membrane. The influence of the hydrophobicity for lateral insertion was studied previously (eg. von Heijne (1997) Mol Microbiol. 24, 249-53; Botelho et al. (2013) J Biol Chem. 288, 4792-8; Meier et al. (2005) JCB 70, 881-8). Therefore, we decided not to try this approach for now.

7. Since the result on mitochondrially targeted Get1-Get2 fusion protein was negative and its reason was not investigated further, this part on mito-GET had better be deleted.

Indeed, the mito-GET data do not allow conclusions on whether the GET complex, in principle, could take over the function of Oxa1. The complementation might not have produced respiration-competent *Δoxa1* cells for various reasons. Still, we think that it is important to also show this negative data here. In particular, the mito-GET data is a sort of control, showing that *Δoxa1* cells are not simply repressed by all kinds of polytopic membrane proteins that are targeted to the inner membrane. We therefore decided not to delete this part. However, we added now a Western blot (novel Fig. S2B) showing that the mito-GET protein is present in cells to rule out that it fails to complement because of proteolytic instability.

Reviewer #3 (Chris Meisinger, signs his review): The authors identify in this study an interesting aspect of the evolution of membrane protein insertion systems. Based on the recently identified structural similarities of the core insertases of bacteria, ER and mitochondria (which show only little sequence similarities) they reveal here that these insertases have also functional similarities. Central of this study is a in vivo reconstitution approach in the yeast model where they replace the mitochondrial inner membrane insertase Oxa1 with the core subunits of the ER membrane complex (EMC). Mito targeted EMC can (at least partially) rescue respiratory growth in oxa1 deletion strains indicating that an ER insertase can take over the function of a mitochondrial insertase. Moreover, they show then with elegant import assays that mito-EMC can indeed mediate import of typical Oxa1 substrates in organello. However, the assembly function of Oxa1 for the Atp9 ring can not be complemented, indicating that the actual import process is a more conserved function than the role in assembly.

This is the first study showing that the members of these insertase family can function as protein insertases also outside of their native organelles/environment. This is a very exciting discovery and opens many new aspects for the field for follow up studies.

The study is presented in a very clear and consise manner; the experiments are of high quality and allow to draw the conclusions presented here.

The manuscript is very well suited for a broad audience including the fields of molecular evolution and general protein biogenesis.

We thank Chris Meisinger for his very positive evaluation of our study!

I have a few questions that came up during reading the manuscript that might be clarified prior publication.

- Fig. 1: can the archeal member of this family (DUF106) also complement lack of mitochondrial oxa1?

We followed the suggestion of the referee and expressed two archaeal DUF106 homologs that were codon-optimized for yeast, equipped with an N-terminal presequence of Oxa1 and a C-terminal ribosome binding domain of Oxa1. One was the DUF106 protein from *Methanocaldococcus jannaschii* which was biochemically characterized before and the other from *Lokiarchaeota archaeon* which is closely related to the eukaryotic ancestor that served as origin of the ER complexes.

We found that none of these DUF106 fusions were able to suppress the growth defect of *Δoxa1* cells on glycerol. Since we did not have time to verify the expression or topology of these proteins we decided not to include this experiment into the manuscript but show the results here for inspection by the referee.

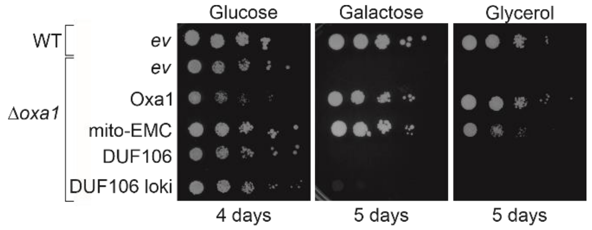

Figure for referee 3
Cells were grown in galactose medium, from which tenfold serial dilutions were dropped onto plates with the respective carbon sources. An empty vector (*ev*) control is shown for comparison. DUF106 and DUF106 loki refer to the versions of *Methanocaldococcus jannaschii* and *Lokiarchaeota archaeon*, respectively

- Could Oxa1 now (the other way around) also complement the EMC complex insertase function? This would be possibly more difficult to test due to the overlap with the Sec61 complex and the lack of a reasonable read out. But maybe there is a way?

This is a very interesting suggestion and we will certainly try this. This suggestion is similar to point 4 of referee 2. Again, we felt that it is not possible to do this experiment convincingly within the time limit of the revision. We also know from a previous study, that targeting of Oxa1 to the ER results in its re-routing to mitochondria and, if this fails, to its degradation by the ERAD pathway (Hansen et al. (2018) Science 361, 1118-1122). Still, we will try this approach in the future, however, we felt that this will be beyond the immediate scope of this manuscript.

- Fig. 4B the authors claim here „Mitochondria containing mito EMC instead of Oxa1 showed no or only low levels of Cox2 precursors". I can not really see this in the Figure. Cox2 seems rather more in the mito-EMC samples. Please clarify.

See also the comment to point 2 of referee 2. In WT, there is basically no Cox2 precursor as insertion occurs very efficiently. In mito-EMC mitochondria, some preCox2 is present,

though much lower levels than in *Δoxa1* mitochondria (compare novel Fig. 4C). We now added additional figure panels (Fig. 4A and E) to show this more clearly.

- how sick is the oxa1 delta strain compared to WT? Would in Figure 2D the growth of WT be faster than of the oxa1 delta strain re-expressing OXA1? This would be interesting to judge the in vivo complementation efficiency (e.g. can the growth rates of left and right graphs be directly compared?). For the in organello experiments (Fig. 3) direct WT comparison was included and looks convincing.

On glucose, WT and *Δoxa1* cells grow with similar rates. On galactose there is a considerable difference, and on glycerol, *Δoxa1* cells are unable to grow (unless complemented with mito-EMC or Oxa1). Yes, the growth rates in the figure can be directly compared as they are all deduced from the same multi-well plate in parallel. Still, we decided to show them separate in order to avoid confusion by the many different traces. For the inspection of the referee, we now add a figure were the growth rates on different carbon sources are plotted. It is also evident that the *Δoxa1* strain expressing Oxa1 from a plasmid grows essentially like wild type again.

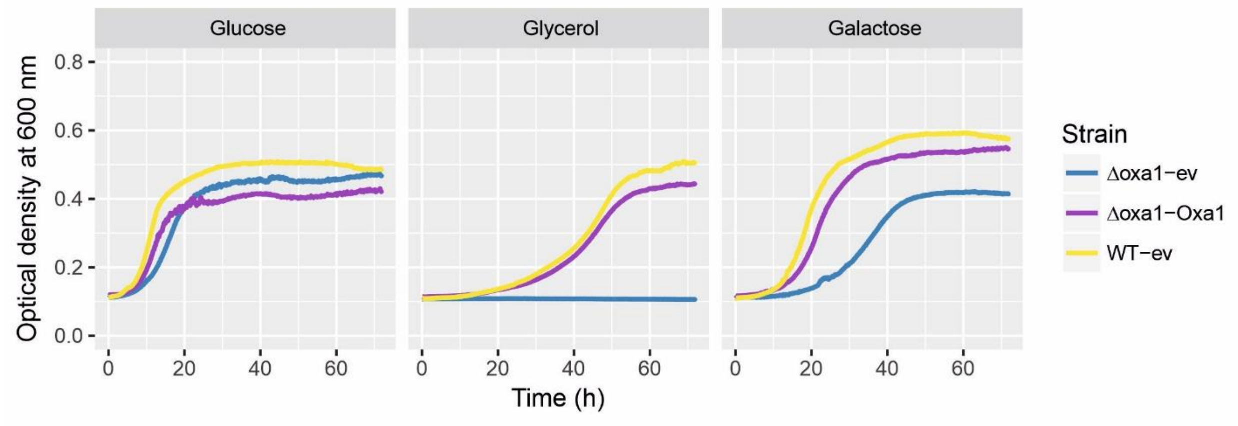

Figure for referee 3

The indicated yeast strains were grown in synthetic media containing the three different carbon sources. Cell densities were measured in parallel in a multi-well plate photometer upon constant shaking of the cultures at 30°C. Shown are mean values of three measurements each.

- For the in organello import experiments (Fig. 3) only one timepoint was used and the topology was then tested (which looks convincing). But did the authors also tested and compared the kinetics (different time points) of the protein insertion process?

We show different time points in a pulse chase setup in Figure S3. For this experiment we labeled the samples for 30 min, then stopped labeling by addition of an excess of cold methionine, followed by a post-incubation (chase) for different times. We added now a quantification showing that the accumulation of the Atp9 oligomer in the mito-EMC sample is strongly reduced at all time points (novel Fig. S3C). We also added a graphical sketch to illustrate how this experiment was performed (novel Fig. S3A).
