# Supplementary material for: The ER membrane complex (EMC) can functionally replace the Oxa1 insertase in mitochondria
Source: PLoS Biol. 2022 Mar 1;20(3):e3001380. doi: 10.1371/journal.pbio.3001380 (PMC8887752; doi:10.1371/journal.pbio.3001380)
Supplement: Attachment — Submitted filename: Response_r2.pdf [file pbio.3001380.s010.pdf]

Point-by-point response

(A) We reduced the number of figures to 4 to stay within the format of a Short Report

(B) We made sure that we provided all grant numbers in the online submission form

(C) We added original file information on all data shown in the study. We chose to present all quantitative data as one supplemental file S1_Data.xlsx.

(D) We added corresponding statements on where the quantitative data information can be found into the text of the manuscript to the panels of Figs 2D, 3D, 4H and S3C.

(E) We added a compiled data set which includes all original images for our study as S2_Raw_Images.pdf. In addition to the scans of the blots and gels, we included pictures for all yeast agar plates shown in the study.
